# Advances in Diagnosis and Management of Pompe Disease

**DOI:** 10.34763/jmotherandchild.20202402si.2001.000002

**Published:** 2020-10-02

**Authors:** James E. Davison

**Affiliations:** 1 Metabolic Medicine, Great Ormond Street Hospital for Children, NHS Foundation Trust, London, UK

**Keywords:** Pompe disease, enzyme replacement therapy, gene therapy

## Abstract

Pompe disease is an autosomal recessive lysosomal glycogen storage disorder caused by the deficiency of acid alpha-glucosidase and subsequent progressive glycogen accumulation due to mutations in the *GAA* gene. Pompe disease manifests with a broad spectrum of disease severity, ranging from severe infantile-onset diseases such as hypotonia and hypertrophic cardiomyopathy to late-onset diseases such as myopathy and respiratory compromise. The diagnosis requires demonstration of deficiency of the lysosomal acid alpha-glucosidase enzyme, which can be assayed in dried blood spot or liquid blood samples, together with supportive biomarker tests, and confirmed with molecular genetic analysis. Targeted screening of at-risk populations and universal newborn screening can result in earlier diagnosis and enable earlier treatment initiation, which result in the potential improvement of clinical outcomes. Disease-modifying treatment with enzyme replacement therapy has partially altered the natural history of the disease, but more efficacious novel therapies are under evaluation including second-generation enzyme replacement therapies, molecular chaperones and gene therapy approaches. Long-term survivors with Pompe disease are now manifesting novel aspects of the disease including widespread vascular disease, smooth muscle and central nervous system involvement, and these emerging phenotypes will require additional specific therapeutic approaches.

## Introduction

Pompe disease (MIM # 232300) is an autosomal recessive lysosomal glycogen storage disorder first described in 1932 (1) and it is caused by biallelic mutations in the *GAA* gene (MIM 606800) that encodes the lysosomal enzyme acid alpha-1,4-glucosidase (GAA; EC 3.2.1.20), also known as acid maltase. GAA has a cellular housekeeping function in the lysosomal degradation of glycogen. The deficiency of GAA results in progressive storage and accumulation of glycogen, initially within the lysosome but subsequently within the cytoplasm and into muscle inter-fibre free glycogen pools causing severe damage to the normal muscle ultrastructure and its function (2). While Pompe disease is predominantly a disorder of muscle affecting both skeletal and cardiac muscle, there are emerging non-muscle phenotypes with other tissues getting affected, which include smooth muscle of gastrointestinal tract and blood vessels, bone, and peripheral nervous system and central nervous system (CNS), resulting in multiple novel manifestations (3).

Pompe disease consists of a spectrum of phenotypic diseases ranging from severe infantile-onset Pompe disease that manifests in early infancy with hypotonia, respiratory insufficiency and hypertrophic cardiomyopathy and natural history of rapid progression with death occurring at 12 months (4) to late-onset Pompe disease that can manifest with myopathy affecting gait and mobility and respiratory decline as late as 7th or 8th decade of life, but without significant cardiomyopathy (5, 6). Pompe disease may be diagnosed as an incidental or unsuspected finding during the investigation of “hyper-creatine kinase-aemia” or other unexplained elevated serum enzymes including lactate dehydrogenase or the ‘liver enzymes’ aspartate/alanine aminotransferase that are liberated from the damaged muscle tissue (7). The clinical spectrum reflects the degree of any residual GAA enzyme activity, and accordingly the combination of severe or less severe mutations occurs leading to a relatively strong genotype–phenotype correlation (8, 9).

## Diagnostics

The cornerstone of the diagnosis of Pompe disease remains to be enzymology, which demonstrates a deficiency in the activity of the lysosomal GAA. Historically this test was conducted in fresh muscle samples or cultured skin fibroblasts, but with minimally invasive testing on lymphocyte or leucocyte (liquid blood) assays or in dried blood spot samples in the first line investigation (10, 11). The assay in leucocytes and dried blood spot samples is complicated by interference from non-lysosomal maltase glucoamylase, and so it requires the use of inhibitors such as acarbose and assays at neutral and acidic pH to derive the required test sensitivity and specificity. The finding of abnormal enzyme activity on the blood spot assay requires second-tier confirmation using enzymology in another sample type, supportive assays and molecular genetic testing. The enzyme diagnosis can be supported by histological findings on examination of a blood film for periodic acid-Schiff stain (PAS)-positive vacuolated lymphocytes (12, 13) and by elevated urine glucose tetrasaccharide levels (14). Molecular genetic analysis for mutations in *GAA* will confirm the diagnosis. There are more than 500 described mutations, with some frequent alleles including the IVS splice site mutation c.-32-13T>G associated with late-onset disease (15), a common Taiwanese mutation (resulting in p.Asp645Glu) (16) and p.Arg854X frequent in African/African-American populations.

Patients with severe infantile-onset Pompe disease can be differentiated into two groups: one group with detectable GAA protein (Cross Reactive Immunologic Material Positive, CRIM+) and another group without any detectable GAA protein (Cross Reactive Immunologic Material Negative, CRIM−). The CRIM− cohort has a worse prognosis and response to treatment with enzyme replacement therapy, which is at least in part due to the generation of high titre anti-drug antibodies by the patient’s immune system that was not exposed to the GAA protein during early immune tolerisation (17). This necessitates modifications to treatment. The CRIM status can be determined by prediction from the genotype if it is known (18) or from a rapid-turnaround blood-based assay in leucocytes (19). Diagnostic algorithms for infantile (20) and late (21) onset Pompe diseases have been established.

### Early Diagnosis

There is emerging evidence that early diagnosis and initiation of treatment result in an improved clinical outcome (22, 23, 24). The ready availability of a simple screening test, i.e. dried blood spot testing, means that any at-risk patient with any potential ’red flag’ signs that could suggest Pompe disease may be tested. This will include all hypotonic infants especially if they also have cardiomyopathy, children with unexplained motor delay and older patients with exertional myalgia, fatigue and also those with unexplained elevated ‘liver enzymes’ or serum CK, those with a limb-girdle syndrome or unexplained respiratory insufficiency.

Also, targeting such ‘at risk’ populations, universal newborn screening (NBS) for Pompe disease has been initiated in several countries (25). Diagnosis from NBS enables identification of patients with infantile-onset Pompe disease, and rapid initiation of early treatment including immunosuppressive treatment for CRIM− patients. After the detection from NBS programs, patients are treated and they show improved long-term clinical outcomes with significantly improved overall and ventilator-free survival, albeit in a population of entirely CRIM+ patients (26). However, the identification of late-onset (or very late-onset) Pompe disease patients from NBS is problematic, raising questions around treatment initiation, and impact on health/life insurance. Protocols for managing patients identified from NBS have been proposed (27, 28).

### Treatment

The management of a patient with Pompe disease requires an extensive multidisciplinary team to address the multisystem manifestations, including cardiology, respiratory, speech and language (swallow), physiotherapy/neurology, genetics and metabolic physicians. Many patients require mobility support and many need non-invasive respiratory support.

Pompe disease is a progressive disorder, but disease-modifying treatments using enzyme replacement therapy are now common in clinical use. Enzyme replacement therapies for lysosomal storage disorder depend on the cross-correction principle, with the uptake of circulating enzyme protein into cells and trafficking to the lysosome via the mannose-6-phosphate signalling system (29). The first human patient was treated with recombinant enzyme replacement 20 years ago (30), and subsequent phase I/II studies (31) led to the market authorisation of alglucosidase alfa (Myzoyme ®, Genzyme) in 2006. Subsequent studies have confirmed the effect of this treatment ameliorating the natural history of infantile-onset (32) and late-onset (33, 34) Pompe diseases.

However, current enzyme replacement therapy is not curative. Patients still display a significant burden of morbidity mandating improvements in current disease-modifying treatment, and novel emerging aspects of the long-term phenotype in patients receiving enzyme replacement therapy require new approaches to address these issues. Factors that are known to affect the outcome and efficacy of enzyme replacement therapy include the CRIM status of infantile-onset patients, the generation of anti-drug antibodies, the degree of (irreversible) muscle damage when treatment is initiated, and concordantly the age and clinical status at the start of treatment (32).

### Novel Disease-Modifying Therapies

Several approaches to improve the efficacy of treatment are under investigation. Increased dose and frequency of enzyme replacement therapy appear to increase efficacy, with some centres using 40 mg/kg weekly dosing of alglucosidase alfa for infantile-onset Pompe disease (Van den Hout, personal communication). For CRIM− infantile-onset Pompe disease patients, immunomodulation at the time of enzyme replacement therapy initiation has been shown to decrease the generation of high-titre anti-drug antibodies and improve the clinical outcomes (35, 36) and necessitates the determination of CRIM status in all newly diagnosed infantile-onset Pompe disease patients. Approaches to increasing muscle uptake of the recombinant enzyme have included exercise regimens during infusions and the use of beta-agonists (37, 38). Several next-generation enzyme replacement therapies are being evaluated that aim to improve muscle-specific uptake by a range of molecular mechanisms, such as increased mannose-6-phosphate tagging or utilising other uptake mechanisms, including in both late-onset [e.g. clinical trials (see (39) for details of trials) NCT02898753, NCT01230801, NCT02782741] and infantile-onset (NCT03019406) cohorts, as well as studies evaluating chaperone therapies in conjunction with highly targeted intravenous enzyme replacement therapies (NCT02675465/NCT04138277).

All enzyme replacement therapies require repeated life-long dosing, and so gene therapy approaches are an effective alternative treatment method. These aim to introduce functioning copies of the *GAA* gene that can be transcribed and translated to produce the GAA enzyme continuously, which can then undergo the normal post-translation modification and processing and trafficking to the lysosome. Approaches include targeting the liver with AAV-vector carried *GAA* gene, utilising the liver as an ‘enzyme factory’ (40, 41) in phase I trial in late-onset patients (NCT03533673), or directly targeting muscle tissues (42) (e.g. NCT02240407).

### Monitoring Disease Progression and Treatment Efficacy

The long-term monitoring of patients with Pompe disease to evaluate treatment efficacy and identify unmet medical needs requires serial multisystem evaluations. This will include measures of cardiac function, respiratory parameters including pulmonary function tests, polysomnography and exercise-tests to evaluate overall function. Swallow function in those with infantile and progressive late-onset Pompe diseases must be evaluated with serial assessments (43).

Assessing the progression of subtle muscle disease in patients with late-onset Pompe disease is important, and several functional assessments are routinely carried out, which include muscle strength (manometry) and detailed neuromuscular scales. Additional monitoring with quantitative muscle MRI (T1 weighted imaging) or Dixon scale is useful (44). Novel biomarkers of muscle progression are being evaluated, including microRNAs (miRNAs), which are small non-coding RNAs having roles in modifying gene expression (45); in particular the dystromirs miR-1-3p, miR-133a-3p and miR-206 that function in muscle regeneration, and the expression levels of these miRNAs correlated significantly with muscle function tests (46).

### Emerging Phenotypes

With long-term survival of patients with infantile-onset Pompe disease, who would have died otherwise in infancy, having a range of emerging Pompe-related phenotypes is becoming apparent. Further, patients with late-onset Pompe disease may have disease manifestations not directly related to skeletal muscle involvement. These additional features must be monitored and addressed, and novel therapeutic approaches will be required to provide holistic disease-modifying treatment.

While hypertrophic cardiomyopathy is the prominent cardiac manifestation of Pompe disease in those with the infantile-onset disease, cardiac conduction defects are recognised in long-term survivors including supraventricular tachycardias and Wolf-Parkinson-White syndrome. Patients with late-onset Pompe disease are also at risk of abnormal cardiac rhythms, short PR-interval and repolarisation abnormalities (47, 48, 49).

Involvement of bulbar muscles together with macroglossia contributes to dysphagia, and many patients especially with the infantile-onset disease require enteral tube feeding (43). Gastrointestinal disturbance due to smooth muscle dysfunction is common, manifesting with diarrhoea, and pelvic muscle weakness may result in urinary and faecal incontinence. (50, 51).

Bone involvement with the risk of osteopenia/osteoporosis (potentially secondary to impaired mobility), evolution of secondary kyphoscoliosis (myopathy) and chronic pain is well recognised and should be treated symptomatically (52).

The smooth muscle of blood vessels can also be affected with glycogen accumulation, and there is emerging evidence of increased risk of intracerebral arterial aneurysms and other vascular defects in patients with Pompe disease, with some 3% of late-onset patients dying from cerebral aneurysmal rupture (48, 53, 54). Baseline cerebral magnetic resonance angiography is indicated in all patients.

Although there is little normal glycogen storage within the CNS, there is some normal expression of GAA within the CNS and consequently there is slow accumulation of glycogen in patients with little or no residual GAA activity (55). Thus, there is evidence of primary CNS involvement in patients with infantile-onset Pompe disease, which manifests as progressive abnormalities on MRI brain imaging (56), sensorineural hearing loss (57, 58) and impaired function with abnormalities of processing speed and emergent learning difficulties (3, 59, 60). There is also evidence of potential cognitive dysfunction in patients with late-onset Pompe disease (61) although these patients do not seem to have the same progressive white matter disease as seen in the infantile cohort, probably reflecting the degree of residual enzyme activity and multi-factorial causes for the CNS disease(62). Conventional intravenous enzyme replacement therapies and the therapies that are presently under development are not expected to cross the blood–brain barrier and are therefore unlikely to be effective in ameliorating the CNS component of Pompe disease. Similarly, gene therapies that aim to target muscles or utilise the liver as a production source of the enzyme may not be effective in treating the brain, and so CNS-targeted therapies are must for effective treatment (63).

## Conclusion

Rapid progress has been made in the understanding of the pathophysiology of Pompe disease, and exciting developments in disease-modifying therapies are under evaluation. An earlier diagnosis may be facilitated by targeted or universal screening, but appropriate guidelines for the monitoring and management, including specific therapy initiation, of patients identified through such screening is required. New approaches to evaluating novel therapies, including methods such as quantitative MRI or biomarkers such as miRNAs for evaluating serial changes in muscle function may guide future therapeutic protocols. The emergence of novel phenotypic aspects of Pompe disease in long-term survivors who are treated with first-generation enzyme replacement therapy requires appropriate monitoring and further development of tissue-specific targeted supportive and disease-modifying therapies.
